# Criminal behavior and contingency

**DOI:** 10.3389/fpsyg.2023.1209619

**Published:** 2023-08-03

**Authors:** Chang-Moo Lee

**Affiliations:** Department of Industrial Security, Chung-Ang University, Seoul, Republic of Korea

**Keywords:** causes of crime, contingency, criminal behavior, criminological theories, determinism

## Abstract

Although their perspectives and approaches vary, existing criminological theories are all based on the deterministic optimism that the crucial causes of criminal behavior must exist and can be uncovered. However, no key factor can fully explain the causes of criminal behavior. All factors that directly affect the occurrence of criminal behavior are important, and contingency is always at work. More feasible crime prevention and control measures can be proposed only considering the contingency factor. The aim of this study is to point out the limitations of the deterministic view of existing criminological theories that explain the causes of crime after knowing the results, and simultaneously to propose the contingency model with viable alternative solutions.

## Introduction

Determining the causes of criminal behavior is a fundamental issue in criminology. Various disciplines have explored this topic, from the philosophical perspective of classical theories and positivism to the fields of biology, psychology, and sociology. These efforts have also included interdisciplinary and integrated approaches. Despite their differences, all approaches share a deterministic optimism that they can uncover the root causes of criminal behavior, leading also to the conclusion that crime prediction is possible. The assumption of the existence of key elements of criminal behavior is based on the causal theory, according to which there is an effect because there is a cause. Such a belief seems possible because the causes of crime are analyzed as the result of a criminal behavior that has already occurred.

The analysis of a certain behavior can only be accurate for that specific case. This causal belief can be considered as an outcome bias and a narrative fallacy in terms of behavioral economics (Taleb, 2001; Kahneman, 2011); that is, it involves analyzing and explaining the causes of a behavior in accordance with the theoretical framework while knowing the consequences and all the circumstances. Thus, the deterministic analysis of the causes of criminal behavior is necessarily problematic. Even when various conditions for the occurrence of criminal behavior are met, the fulfillment of these conditions does not necessarily lead to crime; that is, low self-control, social learning, strain, neutralization of criminal behavior, criminal opportunity, and other conditions for criminal behavior do not necessarily lead to crime.

The conditions for criminal behavior proposed by existing criminological theories are certainly related to crime and are necessary conditions for criminal behavior to occur. However, they are not sufficient conditions. Therefore, existing theories cannot explain all criminal behaviors and has theoretical limitations, although they are logically plausible and empirically valid.

The reason criminal behavior does not necessarily occur, even when the elements for causing criminal behavior are given, is because there are unpredictable factors, such as “contingency.” Even at this moment, everything is changing. As the same conditions cannot exist in the world, the same criminal behavior does not exist, either. That is why criminals do not always commit crimes, no matter how much they are brimming with criminality or how many criminal opportunities there are. This is also why criminal behavior cannot be accurately predicted.

Thus, the purpose of this study is to point out the problem of the hindsight fallacy, which explains causes in the state of knowing the outcome, based on the deterministic optimism that the causes of criminal behavior can be accurately identified. Furthermore, this study aims to answer to the question: “Why does criminal behavior occur?” This study focuses on criminal behavior, not crime itself; that is, it addresses the issues of why and how behaviors that have already been defined as crimes occur.

## Deterministic views of criminal behavior

Most of theories to explain the causes of criminal behavior are based on deterministic causality, regardless of the different approaches (Gottfredson and Hirshi, 1987; Akers, 1994; Miethe and Meier, 1994; Agnew, 1995; Mannon, 1997; Walsh, 2014; Boutwell et al., 2015; Paternoster, 2017; Winfree and Abadinsky, 2017). These theories focus on the particular variables that contribute to crime (Agnew and Messner, 2015). They are designed to show that the causes of criminal behavior lie in the body, mind, and social relationships, meaning that criminal behaviors are caused by important biological, psychological, and sociological determinants. Even theories of the behavior of criminal law take the deterministic position that criminal behaviors are “determined primarily by how the law is written and enforced” (Vold et al., 1998, p. 11).

Although there are many criminological theorists who accept soft determinism or indeterminism, the deterministic model is dominant in individual-level criminological research (Agnew, 1995). Mannon (1997) claims that much individual-level criminological research is based on deterministic models. Agnew (2016) asserts that the three major criminological theories, including strain theory, social learning theory, and control theory, are also based on deterministic causality (Agnew, 2016).

As argued, most criminological theories are based on causation, which appears to be a deterministic concept (Frosch and Johnson-Laird, 2011). Matza (1964) explains that such deterministic view, like other phenomena in the natural world, is due to the epistemological standpoint of positivist criminology that human behavior is subject to deterministic laws. Human behavior, such as crime, is caused by internal or external forces acting on individuals (Matza, 1964).

The life course or developmental approach is also deterministic in that it attempts to find the root of crime (Lilly et al., 2015). This approach is also optimistic as it assumes that crime can be prevented or controlled by eliminating or reducing crucial factors for criminal behavior in the developmental process.

Attempts to explain the causes of criminal behavior in terms of criminal motivation and opportunity have also been based on a deterministic perspective. Cantor and Land (1985) argue that motivation and opportunity represent “two structural effects of opposite algebraic sign” (p. 321). Felson and Clarke (1998) also argue that all crimes are caused by two factors: the offender’s motivation to commit a crime and the opportunity to perform the desired act in a given situation. However, Miethe and Meier (1994) argue that most criminological theories are theories of criminal motivation and focus on finding the determinants of criminal motivation. Weisburd et al. (2014) and Wilcox and Cullen (2018) also point out that criminological theories focus on individuals and motivation. In contrast, the opportunity thesis posits that “the convergence in time and space of suitable targets and the absence of capable guardians may even lead to large increases in crime rates without necessarily requiring any increase in the structural conditions that motivate individuals to engage in crime” (Cohen and Felson, 1979, p. 589). Situational criminological theories, such as the routine activity theory, the crime pattern theory, the multi-contextual opportunity theory (MCOT), and the situational action theory (SAT) emphasize the importance of criminal opportunity (Cohen and Felson, 1979; Clarke, 1980; Clarke and Felson, 1993; Miethe and Meier, 1994; Wikström, 2014; Wilcox and Cullen, 2018). Because these theories assume that crime occurs when opportunities for crime arise and that crime can be prevented by reducing opportunities (Felson and Clarke, 1998), they do not deviate from the deterministic view.

As for the deterministic perspective, another problem with criminological theory is the tendency to simplify as much as possible to explain all criminal phenomena with as few variables. Various integrated theoretical approaches, including research aimed at integrating different theoretical viewpoints into a single overarching perspective (Boutwell et al., 2015) attempt to achieve generalization through simplification (Gottfredson and Hirschi, 1990; Miethe and Meier, 1994; Felson, 2006; Cullen, 2011; Agnew, 2016; Kavish and Boutwell, 2018; Akers et al., 2020). The idea of a general theory that can explain everything with a single concept must be attractive. Parsimony seems to be the main weapon of optimistic determinism that allows generalization. However, this simplification cannot fully reflect reality. There are bound to be exceptions that the theory cannot explain, which is also the reason for the gap between theory and reality.

## Causality and contingency

The instinctive desire to understand the causes behind an event leads to the search for patterns. Humans are always “too quick to see order and causality in randomness” (Kahneman, 2011, p. 117). Most of the patterns we find have a causal basis. Our survival has been supported by finding patterns with empirical and causal bases.

We could tell that a wild animal was approaching when we heard and saw the grass move in the forest, and we could predict rain when dark clouds appeared in the sky. It was more likely that the forest grass was simply shaken by the wind, but people have been more attracted to causal relationships based on the evolutionary psychology judgment of false positives (Barkow, 2006; Johnson et al., 2013; Huneman, 2014). However, patterns are the result of randomness rather than causality.

Francis Bacon, in the Novum Organum, said, that “once the human intellect has adopted an opinion, it endorses and attracts everything that supports it. Even if there are more cases that conform to the opposing views and their importance is greater, the human intellect ignores them and dismisses them with scorn or some kind of discrimination. Those who take pleasure in this vain pride pay attention to events when they agree with their views. In much more cases of inconsistency, however, it is ignored and overlooked” (Hand, 2014, p. 35). Bacon’s argument shows the seriousness of confirmation bias which does not rely on scientific observations.

Modern scientific laws are deterministic. The so-called “clockwork universe” recognizes that the universe evolves along a well-defined path like a clock (Shapin, 1996; Berryman, 2021). From this perspective, nothing is unpredictable. Unpredictability is simply explained by ignorance of the surrounding conditions or the principles of nature. Albert Einstein said “God does not play dice” arguing that the results of measurements appear as probabilities because we do not know all the variables that govern the behavior of particles, the so-called “hidden variables theory” (Hand, 2014).

In modern quantum physics, however, everyday physical laws do not apply, and all events are determined only by probabilities. The radioactive decay of the quantum world or the transition of atoms simply happens, for no particular reason (Gribbin, 1984). There is no direct causal relationship. As with Heisenberg’s uncertainty principle, the more precisely the position of a particle is known, the less precisely the momentum of the particle is known (Gribbin, 1984). Heisenberg (2009) says that quantum physics has revealed the inherent unpredictability of nature. Quantum effects have broken the deterministic laws of classical mechanics (Heisenberg, 2009). In modern science, contingency is what fundamentally drives nature, and uncertainty lies at the heart of nature (Glynn, 2010; Cartwright, 2016).

However, probability theory of quantum physics is not the same as saying that all possible phenomena occur randomly without any laws in the world. The word “randomness” means that every phenomenon has the same probability, and quantum physics calculates the probability that the particle is found at a specific location and the probability of having a specific momentum, depending on the physical state of the particle. In this regard, instead of the existing term ‘nondeterminism’, it is sometimes referred to as probabilistic determinism in the philosophical world. Classical determinism only acknowledges linear causal relationships in which one event produces only one outcome, and probabilistic determinism accepts causal relationships in tree structures in which one event can have multiple possible outcomes.

In chaos theory, as explained by Edward Lorenz, the present determines the future, but the approximate present does not determine the future approximately (Danfort, 2020). As humans only ever know the present approximately, it is impossible to predict the future (Hand, 2014). The “butterfly effect” noted by Lorenz means that minute differences in initial conditions make the state of a system in the near future completely unpredictable.

From an evolutionary biology perspective, evolutionary change can be thought to be brought about by the deterministic force of natural selection, but natural selection occurs through the action of unpredictable and random factors (Blount et al., 2018; McConwell, 2019). Monod (1971), a Nobel laureate and French molecular biologist, argues that the evolution of life is a product of chance. DNA is also subject to quantum fluctuations and is constantly influenced by the external environment. Mutations are the result of these quantum fluctuations; thus, they are events that occur by chance (Monod, 1971). It is estimated that in humans about 100 million mutations occur in each generation (Monod, 1971), which shows how great the random variability is.

Furthermore, humans are composed of 100 billion neurons, and the interconnection of synapses leads to interactions between these neurons that result in cognitive functions (Ramachandran, 2011). However, because brain cells are constantly evolving, future behavior can never be predicted with the current structure. The principle of uncertainty also applies to brain cells and psychological decisions. In this regard, Harman’s argument that human beings are only a product of chance, the product of an “incalculable number of random events” makes sense (Harman, 2014, p. 490).

The claim that there is only one final explanation for everything is problematic. Mulkay (1981) points out the problems with this deterministic approach because the facts and content underlying the theories that claim that a single definitive approach is possible change depending on the social context and situation. The attempt to establish a general theory is very ambitious and attractive, but for that very reason it fails.

There is hardly any specific, decisive and final explanatory factor that can explain everything. Kuhn (1977) argues that there is no theory that can solve all puzzles at a given time, and that the solutions already obtained are not perfect. On the contrary, it is the incompleteness of the existing data-theory fit that defines many of the puzzles that characterize normal science. If any discrepancy between the data and the theory must be considered a rejection of the theory, then all theories should always be rejected (Kuhn, 1977).

As Krumboltz (2009) states with his “planned happenstance theory,” contingency plays an important role in a person’s career. No one can accurately predict the future. No one knows with certainty “which people one will meet, who will call, or what letters or e-mails will arrive on any given day” (Mitchell et al., 1999, p. 116). It is because of contingency that even brothers born to the same parents take completely different life paths. A person’s life process is influenced by unpredictable events (Mitchell et al., 1999).

Yu et al. (2020) also argue that there is no single strategic option that fits all. They argue that both internal and external dimensions have an impact on shaping an organization’s strategy and that no single model can provide optimal results. Therefore, it is necessary to consider the contingent relationship between the independent variable and the dependent variable. The circumstance-contingent factors could influence the effects of the independent variable (Yu et al., 2020). Contingency exists when A cannot prove that it necessarily results in B, when there is an exception, and when the cause is too complex and cannot be specified, in the sense of the principle of a complex system. Correlation, on the other hand, indicates the statistical association between variables by measuring the extent to which they are related or covariant. While correlation can reveal statistical significance and probability, estimating contingency proves challenging due to the complexity and numerous variables that elude human comprehension.

## Criminal behavior and contingency

Many criminological theories, including the social learning theory, the self-control theory, and the life course theory, emphasize the importance of early socialization in the development of criminality (Gottfredson and Hirschi, 1990; Sampson and Laub, 1993; Akers, 1998). These theories particularly emphasize the importance of parents. The problem is that having good parents is entirely the result of contingency. Even for individuals with the same parents, their own specific combination of genes is a result of contingency. No one knows in advance one’s genetic characteristics. The time and place of birth are also closely related to the occurrence of crime; however, this is a result of contingency as well.

As Hirtenlehner and Kunz (2017) explain, low self-control has been shown to be the most reliable predictor of deviant and criminal behavior. However, even people with low self-control can live a lifetime completely independent of crime. Conversely, some terrorists may commit criminal acts while exhibiting high self-control.

Criminal motivation and opportunity are considered necessary conditions for crime to occur. However, it is difficult to view criminal motivation and opportunity as deterministic factors for criminal behavior. No matter how great the criminal motivation, people are not obsessed with crime all day. Criminal motivation is an independent variable in which individual disposition plays a role, but individual disposition is not stable and is flexible depending on the situation. Contingency matters. There can be no general criminal motivation that applies always and to everyone, regardless of contingent circumstances.

Horney (2006) also criticizes that many criminological theories are problematic in their search for general causes and motives for crime, even though criminal motivation can vary depending on the situation. Violent offenders who commit violent crimes are not always impulsive or antisocial. Mischel and Shoda (1995) studied how people’s behavior changes in different specific situations and found that there is no constant factor. Focquaert (2019) also refutes the one-way causal relationship, noting that many individuals with biological risk factors may not commit crimes, while some individuals who do not have a specific risk factor may commit crimes.

Criminal opportunity can be explained from a psychological perspective. The evaluation and assessment of opportunity may vary from person to person. What is not perceived as an opportunity by others may be judged as a good criminal opportunity by criminals. This is because people intuitively judge the values and costs of actions and do not process information objectively and perfectly as computers do (Ward et al., 2006). Opportunities are created by chance. It is an ex-post explanation that a crime occurred because a criminal opportunity was present, and it is only a narrative fallacy. It is important to pay attention to how criminal opportunities come about.

If situation can be understood as a concept similar to contingency, as mentioned above, the concept of contingency can be explained by situational theories of crime. However, situational theories cannot account for all possible circumstances. If all situations cannot be accounted for, contingency must work. Therefore, situational theories of crime cannot fully explain the causes of all criminal behavior.

A contingency does not need to occur, whereas a necessity must occur. The deterministic causes of crime as asserted by existing criminological theories do not necessarily lead to crime. Low self-control or strain do not necessarily lead to crime. Crime does not necessarily occur even when the conditions for the causes of crime asserted by these criminological theories, including biological, psychological, and sociological theories, are met because contingency intervenes. Therefore, contingency is important in explaining criminal behavior. Although certain factors may be considered the most important factor in the occurrence and prediction of criminal behavior, the reliability and validity of these factors is inevitably reduced when the variable of contingency is interposed.

It is certainly not easy to analyze and predict criminal behavior at both the macro and individual levels. In the 1990s, many criminologists, including Wilson and Petersilia (1995), predicted a sharp increase in crime after the mid-1990s, but crimes actually declined (Zimring, 2007). Wilson and Petersilia (1995) predicted that violent crime would continue to increase in the late 1990s and even warned of “get ready.” The increase in crime was expected based on several factors, such as economic indicators that are evaluated as related to crime rates, but the actual crime rate declined. Since then, several research papers have been published explaining the reasons for the decline in crime; however, they are affected not only by a hindsight bias but also by a narrative fallacy (Pinker, 2011). This is because we cannot accurately predict the criminal situation 1 year or 5 years in the future. The contingency of criminal behavior becomes clear when Hand’s (2014) law of necessity is applied to criminal behavior. According to Hand (2014), if a complete list of all possible outcomes can be generated, one of the outcomes will inevitably occur. However, in the case of criminal behavior, a complete list of all possible outcomes cannot be generated. Therefore, criminal behavior is subject to contingency.

Of course, some criminological theories acknowledge the contingency of criminal behavior (Felson, 1998, 2006; Wikström, 2014; Gottfredson and Hirschi, 2020). Gottfredson and Hirschi (2020) argue that “most criminal behaviors are highly opportunistic, momentary, or adventitious (p. 12).” Felson (2006) also argues that “diverse cues are emitted in the course of life” and that some of them lead people to commit crimes (p. 15). However, he does not mention the role and importance of contingency. Criminal behavior is caused by a combination of various factors. Existing theories state that certain factors are more relevant to criminal behavior. Conversely, there is little consideration of the role and importance of contingency in influencing criminal behavior. Positivism, which forms the basis of modern criminological theories, assumes that humans also follow deterministic laws of nature. However, these theories do not seem to consider that contingency and uncertainty fundamentally govern nature, as recognized by modern science such as quantum physics and molecular biology.

Just as water boils only when it exceeds the boiling point, a crime occurs when all the necessary conditions for a crime to occur are met. There should be both necessary and sufficient conditions for criminal behavior to occur. The “chemistry for crime” should presuppose all the necessary conditions, just as “chemical reactions occur only when all of necessary ingredients are mixed together” (Felson, 1998, p. 52).

The perfect conditions for crime are never the same and they are constantly changing. Whether or not self-control is present, the same person will react differently to the same stimulus depending on differences in time and space. Even in the same person, the crime depends on the subtle difference in location or the difference of only a few seconds.

Henri Poincare, a French mathematician and physicist, emphasizes the importance of contingency, saying that a series of small causes can combine to produce a huge effect, and that a small cause can determine a large effect (Pinker, 2011). Even when all other conditions are equal, a very small difference will result in a different response. Small differences can change the end result (Thaler and Sunstein, 2008). With respect to crime, small differences also lead to differences in criminal behavior. As illustrated by the butterfly effect of chaos theory, a butterfly in Brazil can cause a tornado in Texas. The principle of criticality, where the last drop of water breaks the bank, is at work here.

At a certain point, people are similar to uranium atoms. The uranium explosion does not require a large change in mass (ΔE = Δm × c^2^). Small changes in mass can lead to massive explosions through chain reactions. Indeed, very small provocations and excitations often lead to various crimes, including assault. Conversely, even extreme provocations and arousals do not necessarily lead to criminal behavior if only very small factors intervene. People do not always react the same way, but differently depending on the situation, which is determined by contingency. Self-control is a psychological judgment and decision that always changes depending on the situation. This is because the human mind does not remain the same due to countless variables that humans cannot control.

According to Hand’s (2014) law of probability leverage, a small change in conditions can have a large effect on probability. As it is impossible to control all criminal circumstances or variables, even a small difference determines whether a criminal behavior occurs. The conditions of occurrence are determined by chance. Thus, it can be said that the sufficient condition for the occurrence of criminal behavior is chance. Assuming that there are 100 factors that directly affect the occurrence of a crime, a crime will not occur if even one of them is absent or altered.

Genetic factors, personality disorders, social disorganization, differential association, strain, self-control, social learning, labeling, neutralization, conflict, and optimism bias are not deterministic factors that inevitably lead to criminal behavior. All these factors are important determinants of the occurrence of criminal behavior, but crime will not occur if some factors are absent or altered, no matter how minor they are.

Various factors act sequentially and simultaneously to cause criminal behavior. Everything in the world is intertwined with everything through quantum signal exchange (Mindell, 2000). As Jung (2010) asserts in explaining synchronicity, it can be a “meaningful coincidence” between events that do not appear to have a causal relationship.

The occurrence of criminal behavior depends ultimately on the offender’s choice and decision; however, countless factors influence the final decision and action. Even minor changes can affect the occurrence of criminal behavior. Trying to explain complex phenomena such as crime with a small number of key variables makes it difficult to offer an accurate and predictive analysis. That is why it is important to consider the relationships between as many variables and factors as possible. Even though the key factor in the occurrence of criminal behavior is not necessarily connected to criminal behavior, considering as many variables as possible related to crime is a way to increase the accuracy and probability of analyzing and predicting the cause of criminal behavior. It can also be a way of preventing and controlling crimes, lowering their probabilities. This is because criminal behavior, like all phenomena in the physical world, is ultimately a product of contingency.

## Conclusion

Every event in the world must be the first. There is no such thing as the exact same event. Crime is not an exception. There are no criminal cases that resemble those of the past. Therefore, it is impossible to predict anything with 100% accuracy. Criminal behavior is a product of contingency; it occurs when all criminal conditions are met by contingency. The claim that crime is a product of contingency does not mean that all efforts to prevent crime are useless. Existing theories also do not claim that all crime can be prevented or reduced by controlling each key factor, but rather that they increase the feasibility of crime prevention and control. However, this study suggests that even this feasibility should be affected by contingency. It also suggests that there is no deterministic key factor in the occurrence of criminal behavior. This is because too many factors are involved in the actual occurrence of criminal behavior.

As these factors lead to criminal behavior sequentially and simultaneously, criminal behavior can be prevented by changing only a few factors that directly affect its occurrence. To prevent the occurrence of criminal behavior, attention should be paid to the small differences that can avert the tipping points that lead to the occurrence of criminal behavior. To this end, a more accurate analysis and prediction of the cause of criminal behavior can be achieved by listing as many variables as possible that are related to the cause of criminal behavior and analyzing the relationships rather than simplifying the cause of the crime. More accurate analysis and prediction is possible when tens of thousands of variables are included in the analysis rather than just a few key variables.

Analysis techniques that leverage the latest technologies such as big data, AI (artificial intelligence), and quantum computing can support this approach. In addition, approaches such as “contingency planning” that consider the factor of chance can also be proposed as effective crime prevention and control measures. Particularly, modern AI systems are capable of effectively analyzing and predicting criminal behavior by processing extensive amounts of data and identifying patterns, correlations, and risk factors. AI can facilitate the creation of models that take into account various factors and offer valuable insights to assist in decision-making, risk assessment, and resource allocation. Nevertheless, it is crucial to ensure ethical implementation of these systems, incorporating human oversight to guarantee proper consideration of privacy, fairness, and accountability concerns. This oversight is necessary to prevent unjust biases, protect individual rights, and address any potential issues that may arise.

In summary, there are no deterministic elements that necessarily lead to the occurrence of criminal behavior. There is no universal key that opens all doors. Existing criminological theories have their limitations in that they focus on finding these keys. They have the problem of narrative fallacy, which presents the key factors after knowing the results. Therefore, the existing theories do not fully explain criminal behavior, although they have logical probability and empirical validity. The loophole in the existing theories is contingency.

Contingency affects the occurrence of criminal behaviors anytime and anywhere. Therefore, more feasible measures can be proposed for crime prevention and control only when contingency is considered. However, this study has its limitations in that it has no empirical validity through verification and only proposes theoretical hypotheses, which can be criticized as a strawman argument. There is also a problem with contingency arguments avoiding explanation by designating everything that cannot be explained. It can also oversimplify complex phenomena or attach undue importance to certain factors, which seem to be some kind of tautological fallacy. Nevertheless, the contribution of this study is that it emphasized the importance of contingency for understanding of criminal behavior.

## Data availability statement

The original contributions presented in the study are included in the article/supplementary material, further inquiries can be directed to the corresponding author.

## Author contributions

C-ML: conceptualization, writing original draft preparation, and reviewing and editing.

## Funding

This paper was supported by Korea Institute for Advancement of Technology (KIAT) grant funded by the Korea Government (MOTIE) (P0008703, The Competency Development Program for Industry Specialist).

## Conflict of interest

The author declares that the research was conducted in the absence of any commercial or financial relationships that could be construed as a potential conflict of interest.

## Publisher’s note

All claims expressed in this article are solely those of the authors and do not necessarily represent those of their affiliated organizations, or those of the publisher, the editors and the reviewers. Any product that may be evaluated in this article, or claim that may be made by its manufacturer, is not guaranteed or endorsed by the publisher.
